# 

**Published:** 1835-07-01

**Authors:** 


### MEDICAL

### QUARTERLY REVIEW.

---

### No. VIII.

JULY 1,

1835.

---

LONDON:

J. SOUTER, 73, ST. PAUL'S CHURCH-YARD;

SOLD ALSO BY

BURGESS AND HILL; J. CHURCHILL; E. COX; J. HIGHLEY;
RENSHAW AND RUSH, &c.

W. JACKSON, NEW YORK, AGENT FOR THE UNITED STATES.

PRICE FIVE SHILLINGS.

J. AND C. ADLARD, BARTHOLOMEW CLOSE.

